# Developing Pedestrians’ Red-light Violation Behavior Questionnaire (PRVBQ): Assessment of Content Validity and Reliability 

**Published:** 2019-08

**Authors:** Mahdi Moshki, Abdoljavad Khajavi, Homayun Sadeghi-Bazargani, Shahram Vahedi, Saeid Pour Doulati

**Affiliations:** ^*a*^Health Education and Health Promotion Department, School of Health, Social Development and Health Promotion Research Center, Gonabad University of Medical Sciences, Gonabad, Iran.; ^*b*^Community Medicine Department, School of Medicine, Gonabad University of Medical Sciences, Gonabad, Iran.; ^*c*^Road Traffic Injury Research Center, Tabriz University of Medical Sciences, Tabriz, Iran.; ^*d*^Department of Psychology, Faculty of Education and Psychology, University of Tabriz, Tabriz, Iran.; ^*e*^PhD candidate of health promotion, Social Development & Health Promotion Research Center, Gonabad University of Medical Sciences, Gonabad, Iran.

## Abstract

**Background:**

Pedestrians’ unsafe crossing behavior such as not waiting for pedestrians’ red-light expose them at risk of injury, disability, and death. The theory of planned behavior (TPB) is a widely used model used to understand individuals' unsafe behavior for setting interventional goals to prevent injuries.

**Purpose:**

The purpose of this study is to develop a self-completion pedestrians’ red-light violation behavior questionnaire (PRVBQ) based on the TPB and assess the content validity and reliability for this instrument.

**Methods:**

This study was conducted in three phases: 1) PRVBQ development study consists of belief elicitation for item generation and draft instrument construction; 2) Content validity study including face validity of the instrument; and 3) Reliability assessment including internal consistency and test re-test reliability over a 2-week interval. The directed content analysis method used for the analysis of the belief elicitation. The item impact score was calculated as an indicator of quantitative face validity. Content validity index (CVI) in the item level and average scale level, and content validity ratio (CVR) were calculated for quantitative content validity. Intra-class Correlation Coefficient (ICC), and Cronbach’s alpha was calculated for test-retest reliability and internal consistency respectively.

**Results:**

By qualitative belief elicitation study between four to twenty sub-categories were generated, which were classified in the ten categories of the theory of planned behavior: advantages, disadvantages, positive feelings, negative feelings, approving referents, disapproving referents, behaving referents, not-behaving referents, facilitators, and barriers. A primary draft of PRVBQ comprised of 86 items was generated, from which 17 were eliminated due to low face and content validity, remaining 69 item in total. The PRVBQ was rated as having good content validity (individual items CVI ranged from 0.80 to 1, and overall PRVBQ CVI-Average= 0.95, p=0.05). Cronbach’s alpha for the direct measures (reflective indicators) showed that they have excellent internal consistency 0.90. All items showed excellent agreement, ICC= .88 (95% CI [.80, .93]).

**Conclusions:**

This study provided a valid and reliable questionnaire which can be used to predict and to determine their underlying beliefs regarding their red-light violation behavior.

**Keywords:**

Pedestrians, Unsafe road crossing, Validity, Reliability, Questionnaire

